# NF-κB Repressing Factor Inhibits Chemokine Synthesis by Peripheral Blood Mononuclear Cells and Alveolar Macrophages in Active Pulmonary Tuberculosis

**DOI:** 10.1371/journal.pone.0077789

**Published:** 2013-11-04

**Authors:** Kuo-Hsiung Huang, Chun-Hua Wang, Kang-Yun Lee, Shu-Min Lin, Chien-Huang Lin, Han-Pin Kuo

**Affiliations:** 1 Graduate Institute of Medical Sciences, College of Medicine, Taipei Medical University, Taipei, Taiwan; 2 Pulmonary Medicine Research Center, Chang Gung Memorial Hospital, Taipei, Taiwan; University of Leuven, Rega Institute, Belgium

## Abstract

NF-κB repressing factor (NRF) is a transcriptional silencer implicated in the basal silencing of specific NF-κB targeting genes, including iNOS, IFN-β and IL-8/CXCL8. IP-10/CXCL10 and IL-8/CXCL8 are involved in neutrophil and lymphocyte recruitment against *M. tuberculosis* (MTb) and disease progression of pulmonary tuberculosis (TB). Alveolar macrophages (AM) and peripheral blood mononuclear cells (PBMC) were used to study the regulatory role of NRF in pulmonary TB. AM and PBMC were purified from 19 TB patients and 15 normal subjects. To study the underlying mechanism, PBMC were exposed to heated TB bacilli. The regulation role of NRF in IP-10/CXCL10 and IL-8/CXCL8 was determined by NRF knock-down or over-expression. NRF binding capabilities in promoter sites were measured by chromatin immunoprecipitation (ChIP) assay. The levels of IP-10/CXCL10, IL-8/CXCL8 and NRF were significantly higher in AM and PBMC in patients with active TB. NRF played an inhibitory role in IP-10/CXCL10 and IL-8/CXCL8 inductions. We delineate the role of NRF in pulmonary TB, which inhibits the expressions of IP-10/CXCL10 and IL-8/CXCL8 in AM and PBMC of patients with high bacterial load. NRF may serve as an endogenous repressor to prevent robust increase in IP-10/CXCL10 and IL-8/CXCL8 when TB bacterial load is high.

## Introduction

Approximately one third of the world's population is infected with MTb. In most of the infected persons, the host immune response keeps the infection under control. Thus, most infected cases never develop an active disease [1], [2], [3]. However, TB remains the major cause of death due to infectious disease in humans throughout the world. Over the last century TB killed over 100 million people [1], [2], [3]. Therefore, to delineate host factors that control the individual susceptibility to MTb infection is urgent.

After MTb infection, innate immunity initially predominates in the host response. The subsequent recruitment of T lymphocytes to lung is necessary for the containment of MTb within granulomas, which consist of activated macrophages, T lymphocytes, fibroblasts, and epitheloid cells [4]. A complex interaction between different cell populations controls MTb infection and prevents the disease from reactivation. Specific chemokines are released to recruit NK cells, γδ T lymphocytes, and αβ T lymphocytes of CD4^+^ and CD8^+^ phenotypes in sequential order into the site of MTb infection [5], [6]. Increased release of chemokines, including IL-8/CXCL8, IP-10/CXCL10, MIG/CXCL9 and MCP-1/CCL2, has been observed in monocytes, alveolar macrophages and polymorphonuclear granulocytes in patients with pulmonary TB compared to healthy subjects [7], [8], [9].

Enhanced IL-8/CXCL8 release and gene expression in macrophages or monocytes has been shown after exposure to MTb and its components [10], [11]. IL-8/CXCL8 is necessary for granuloma formation [12], limits the growth of intracellular MTb, and enhances the macrophage killing of MTb [13]. In humans, IL-8/CXCL8 gene polymorphism is associated with susceptibility to TB [14], and decreased IL-8/CXCL8 secretion occurs in HIV-infected patients with military TB [15].

IFN-γ-inducible protein 10 (IP-10/CXCL10), a member of the α-chemokine subfamily, is involved in delayed type hypersensitivity [16], attracts monocytes and activated T lymphocytes at inflammatory foci [17], and promotes Th1 responses and IFN-γ gene expression [18]. High levels of IP-10/CXCL10 were detected in the sera of TB patients [19]. Increased number of IP-10/CXCL10-positive cells in bronchoalveolar lavage was significantly increased in TB patients [20]. An increased expression of IP-10/CXCL10 mRNA in the lung was shown in mice following exposure to low-dose aerosols of MTb [21]. In addition to chemotaxis, IP-10/CXCL10 also contributes to the necrosis of tuberculous granulomas by inhibiting angiogenesis [22].

The induction of both IP-10/CXCL10 and IL-8/CXCL8 by MTb is NF-κB dependent [23], [24]. NRF is a transcriptional silencer and is implicated in the basal silencing of specific NF-κB targeting genes, including iNOS, IFN-β and IL-8/CXCL8 [25], [26], [27]. However, the role of NRF in pathogenesis of human diseases has not been explored. We have now demonstrated that NRF is upregulated in the circulating monocytes and AM of patients with active pulmonary TB, and modulates synthesis and release of IP-10/CXCL10 and IL-8/CXCL8. The regulatory mechanism was also delineated.

## Materials and Methods

### Study Populations

Nineteen patients with active pulmonary TB infection and 15 healthy volunteers were recruited. All subjects were non-smokers and HIV negative. For all patients, at least one recent sputum specimen was culture-positive for *M. tuberculosis*. Sputum was collected on 3 consecutive days and the AFB smear was graded as: 0, absence of bacilli; 1, 1 to 9 bacilli; 2, 10 to 29 bacilli; and 3 more than 30 bacilli per 30 oil-immersion fields. The gradings over 3 days were summed as an index of bacterial sputum load [28]. Patients were categorized into two groups: AFB-low group with bacterial load ≤3 and AFB-high group with >3. All the patients in AFB-high group had sputum AFB smear of grade 2 or greater on a single day. In contrast, patients in AFB-low group had sputum AFB smear of grade 1 or less on any single day.

None of the patients were taking corticosteroids, antibiotics or other immunosuppressants within 3 months before entry into the study. Patients in poor nutrition status (body mass <90th percentile or mid-arm circumference and triceps skin fold thickness <25th percentile) were excluded. Patients with systemic or local inflammatory diseases, such as lupus erythromatosus, sepsis, diabetes mellitus, lung cancer, bronchiectasis and interstitial lung diseases were also excluded.

The control group consisted of 15 healthy, sex and age-matched volunteers, who had been BCG vaccinated. None of them had a history of lung disease based on physical and chest radiographic examinations. All subjects were current nonsmokers. None of them had any upper respiratory tract infection within the last 6 weeks. None of them took any antibiotic or chronic medication at the time of evaluation. This study was approved by Chang Gung Memorial Hospital Ethical Committee (CGMH IRB 96-1708B). Written informed consent was obtained from all patients and normal subjects.

### Amplified Mycobacterium Tuberculosis Direct (AMTD) Test

The direct Gen-Probe AMTD test was performed in our laboratories following the procedure of the manufacturer, as our previously described [29]. All the tests were conducted by the same technician, who was certified by a local Gen-Probe dealer to perform the test on clinical samples. AMTD tests were read using a Gen-Probe Leader 50 luminometer following the probe selection step. Each AMTD test run included positive and negative amplification controls and hybridization controls. A positive AMTD test result was defined by Gen-probe as an initial negative result (<30,000 relative light units (RLU)), an initial positive result (≥500,000 RLU), or an initial equivocal result (30,000 to 499,999 RLU). Samples giving results between 30,000–500,000 RLU were repeated to confirm for positivity. A repeat result greater than 30,000 RLU was considered positive.

### Cell preparation and culture

BAL was performed as described previously [28] and lavaged cells were resuspended in RPMI-1640 (GIBCO, Grand Island, NY, USA) supplemented with 10% fetal calf serum (FCS, Flow Laboratories, Paisley, Scotland, UK) at 1×10^6^ cells/ml.

Thirty ml of heparinized blood was collected from normal subjects and TB patients. Peripheral blood mononuclear cell (PBMC) and alveolar macrophages (AM) were isolated on a Ficoll-Hypaque (Sigma, St Louis, MO, USA) density gradient (d = 1.077 g/cm^3^), and were washed three times in RPMI-1640.

Purified PBMC (1×10^6^ cells/ml) were pretreated with or without NF-κB specific inhibitor JSH-23 (Merck KGaA, Darmstadt, Germany) half hour before incubation with or without heated H37-RA MTb (H. TB) (DIFCO) for 6 hours. The culture supernatant was collected and frozen at −70°C before analysis.

Serum and the culture supernatants were collected for assay of IP-10/CXCL10 and IL-8/CXCL8 by commercial ELISA kits (R&D Systems, Minneapolis, MN).

### Quantitative real-time PCR (qPCR)

Total RNA was isolated from cells using TRIzol reagent (Invitrogen, Grand Island, NY) according to the manufacturer's instruction. cDNA was reverse-transcribed from isolated RNA by incubating 200 ng of DNase-treated RNA with the first-strand synthesis kit (Advanced Biotechnologies). qPCR was performed in a LightCycler 2.0 System (Roche Applied Science) using LightCycler DNA Master SYBR Green I (Roche Applied Science). Samples were denatured at 95°C for 10 min, followed by 45 cycles of annealing and extension at 95°C for 15 s, 60°C for 5 s, and 72°C for 10 s. Melting curves were obtained at the end of amplification by cooling the samples to 65°C for 15 s, followed by further cooling to 40°C for 30 s.

Data were analyzed by standard curve method of relative quantification using the LightCycler analysis software.

### Quantification of NF-κB p65 DNA-binding activity (TransAM Assay)

To determine whether basal NF-κB activities were altered in PBMC of TB patients, the NF-κB TransAM kit (Active Motif) was used to measure the levels of p65 and p50 subunit activity in PBMC of TB patients in comparison with that of normal subjects. Briefly, cells nuclear extraction was prepared by using the Nuclear Extract Kit (Active Motif) and protein concentrations were measured using the Bradford assay (Bio-Rad). Lysates (10 µg total proteins) were incubated in ELISA wells coated with the NF-κB consensus site (5′-GGGACTTTCC-3′) recognized by active p65, p65 was then detected using a specific antibody, followed by a secondary antibody conjugated to peroxidase.

### Immunostaining and confocal laser microscopic analysis

AM, or PBMC treated with or without H. TB were spun down on slide then fixed in methanol at −20°C for 5 min. The cells were then blocked with 1% BSA/PBS at room temperature for 30 min and incubated with the rabbit anti human NRF Ab at room temperature for 1 h. After washing, the cells were incubated with a Cy3-conjugated anti-rabbit Ab (Chemicon International) and incubated with Hoechst dye (Sigma-Aldrich). After washing and air-drying, the cells were mounted with anti-fade mounting medium (Dako Cytomation). Images were acquired with a confocal laser-scanning microscope (Leica) and analyzed by Metamorph Image Analysis (Universal Imaging).

### Western Blot Analysis

Total cellular proteins were extracted from PBMC cells by freeze-thawing samples in Reporter lysis buffer (Promega). Proteins were subjected to 7.5% SDS-PAGE and blotted onto nitrocellulose filters. NRF was detected with β-actin (sigma) and an alkaline phosphatase-conjugated anti-mouse secondary Ab (1/100,000 dilution; Calbiochem) or specific anti-NRF Ab and an alkaline phosphatase-conjugated anti-rabbit secondary Ab (1/10,000 dilution; Calbiochem). Blots were incubated with ECL solution (LumiGLO; Amersham Bioscience). Images were acquired and analyzed using G: BOX (Syngene).

### Transfection of siRNA and plasmids

To knockdown NRF overexpression, Si-RNA (Si-Scramble and Si-NRF) was introduced into PBMCs of TB patients. To evaluate whether overexpression of NRF may upregulate the release of IP-10 and IL-8, plasmid DNA (p-CMV and p-CMV-NRF) were introduced into PBMCs of normal subjects (1×10^6^ cells/ml). The transfection was performed by lipofectamine 2000 kit (Invitrogen, Grand Island, NY). After transfection, cells were incubated in complete medium for 48 h (for Si-RNA) or 24 h (for plasmid DNA). The protein of transfected PBMCs from TB patients was harvested for Western blot analysis. PBMCs of normal subjects after transfection were cultured with H. TB for 6 hours and the supernatants were collected for ELISA.

### ChIP assay

ChIP assays were performed as described previously [30]. After stimulation, protein-DNA complexes were cross-linked at 37°C for 10 min by formaldehyde (1% final concentration). Cells were washed with ice-cold PBS then resuspended in 200 μl of SDS lysis buffer (50 mM Tris (pH 8.1), 1% SDS, 5 mM EDTA, and complete proteinase inhibitor mixture) and subjected to five cycles of sonication on ice with 10-s pulses. Sonicated samples were centrifuged to spin down cell debris, then supernatant was diluted by 1.8 ml ChIP dilution buffer. Twenty μl (1%) of sample was kept as input control. The remained diluted solution was precleared by incubating with 80 µl of salmon sperm DNA/protein A-agarose-50% slurry for 30 min at 4°C on a rotator. After centrifuge, 900 µl of the supernatant was immunoprecipitated at 4°C overnight on a rotator by using Abs specific for NRF (5 µg), IgG (Santa Cruz Biotechnology) followed by incubation for 1 h at 4°C with 60 µl of salmon sperm DNA/protein A-agarose-50% slurry. Protein-bound immunoprecipitated DNA (IP-DNA) was sequentially washed with low-salt or high-salt immune complex wash buffers. Immune complexes were eluted twice by adding 250 µl of elution buffer (1% SDS/0.1 M NaHCO3). DNA-protein cross-links were reversed by incubation for 4 h at 65°C in 200 mM NaCl/1% SDS, and proteins were digested by incubation for 1 h at 45°C with 70 µg/ml proteinase K (Sigma-Aldrich). DNA was isolated with phenol/chloroform, precipitated with ethanol/0.3 M NaHCOOH/20 µg of glycogen and was resuspended in 50 µl of nuclease- free water. qPCR was performed with 7 µl of DNA sample for quantification.

### Statistical analysis

Data were expressed as mean ± SE. The data were analyzed with Student's *t* test for paired or unpaired data. For data with uneven variation, the Mann-Whitney U test or Wilcoxon's signed ranks test was used for unpaired or paired data, respectively. Statistical significance of results was determined using prism4 software. A value of p<0.05 was considered statistically significant.

## Results

### Clinical characteristics of study populations

**Figure 1:**
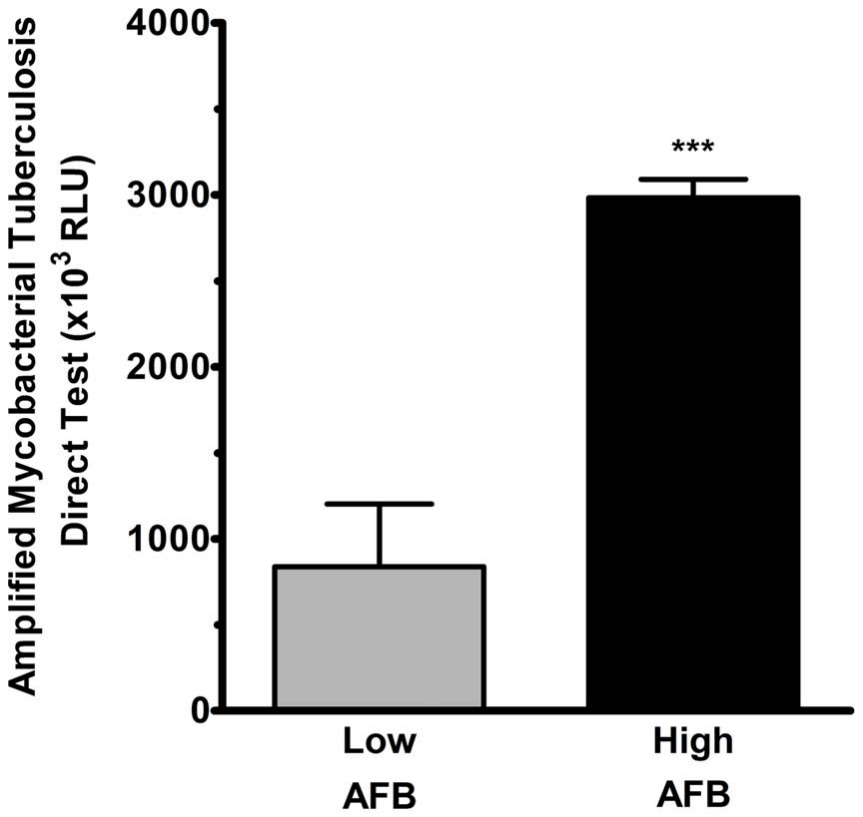
Increased intracellular ribosomal RNA in AM from TB patients. The Amplified Mycobacterium Tuberculosis Direct (AMTD) test that measures the intracellular ribosomal RNA in AM of pulmonary TB patients is significantly higher in patients with high M. TB load of sputum (High AFB, n = 6) than that of patients with low sputum bacterial load (Low AFB, n = 6). Data are mean ± SE. *** p<0.001.

Nineteen patients with active pulmonary TB and 15 normal subjects were recruited in this study. There was no significant difference between TB patients and normal subjects in terms of age, gender or underlying diseases (Table 1). Ten of TB patients were categorized into AFB-low group, 9 were in AFB-high group, determined by the sputum bacterial load. *M. tuberculosis* was detected by the presence of ribosomal RNA in AMs or PBMCs using Amplified Mycobacterium Tuberculosis Direct (AMTD) test. The bacterial loads in AMs in terms of ribosomal RNA were highly compatible with sputum bacterial load of *M. tuberculosis* (Figure 1). However, we did not detect the presence of *M. tuberculosis* ribosomal RNA in peripheral blood mononuclear cells.

**Table 1:** Characteristics of normal subjects and patients with active pulmonary tuberculosis.

	Normal (n = 15)	Tuberculosis (n = 19)		
		Total	AFB-low	AFB-high
Age, years	52.7±4.5	48.6±4.8	42.8±6.7	55.1±6.5
Gender, (F/M)	5/10	5/14	3/7	2/7
Bacterial load	NA	3.4±0.8	0.3±0.2	6.9±0.6^††^
Drug resistance^1^ %			10.0%	
BAL Recovered (ml)	39.7±5.6	36.7±5.2	31.8±4.9	42.1±9.7
Cell count (×10^5^/ml)	1.6±0.3	4.7±1.2*	5.0±2.2*	4.3±0.8*
Viability, %	94.7±1.2	89.2±2.2	92.4±2.0	85.7±3.9
AM, %	91.4±1.2	79.8±3.1**	79.3±5.2*	80.2±3.7*
Lymphocytes, %	6.5±1.2	13.8±3.1*	16.5±5.3*	10.7±2.6*
Neutrophils, %	1.9±0.2	6.3±1.7*	3.9±1.8	9.0±2.7*
AM (pg/ml)				
IP-10/CXCL10	30.0±7.6	950.9±283.0**	215.2±40.4**	1768±468.5**
IL-8/CXCL8	10414±2683	37834±4208**	36980±5387**	38783±6902**
Serum (pg/ml)				
IP-10/CXCL10	62.8±5.4	392.2±70.5**	300.5±54.4**	518.4±143.5**
IL-8/CXCL8	4.5±1.0	31.9±8.5**	41.4±14.6**	21.4±7.1**
PBMC (pg/ml)				
IP-10/CXCL10	29.6±5.5	278.5±54.6**	197.7±36.1**	389.6±112.2**
IL-8/CXCL8	6917±1900	27639±3368**	26505±3982**	29200±6125**

Values presented as mean ± SEM.

*Abbreviations:* AM  =  Alveolar macrophages; NA  =  not available; BAL  =  Bronchoalveolar lavage.

1 Resistance to either isoniazid, rifampin, or streptomycin.

* p<0.05, ** p<0.01 compared with normal subjects. † p<0.05, †† p<0.01 compared with AFB-low.

### Increased release of IP-10/CXCL10 and IL-8/CXCL8 in pulmonary TB

**Figure 2:**
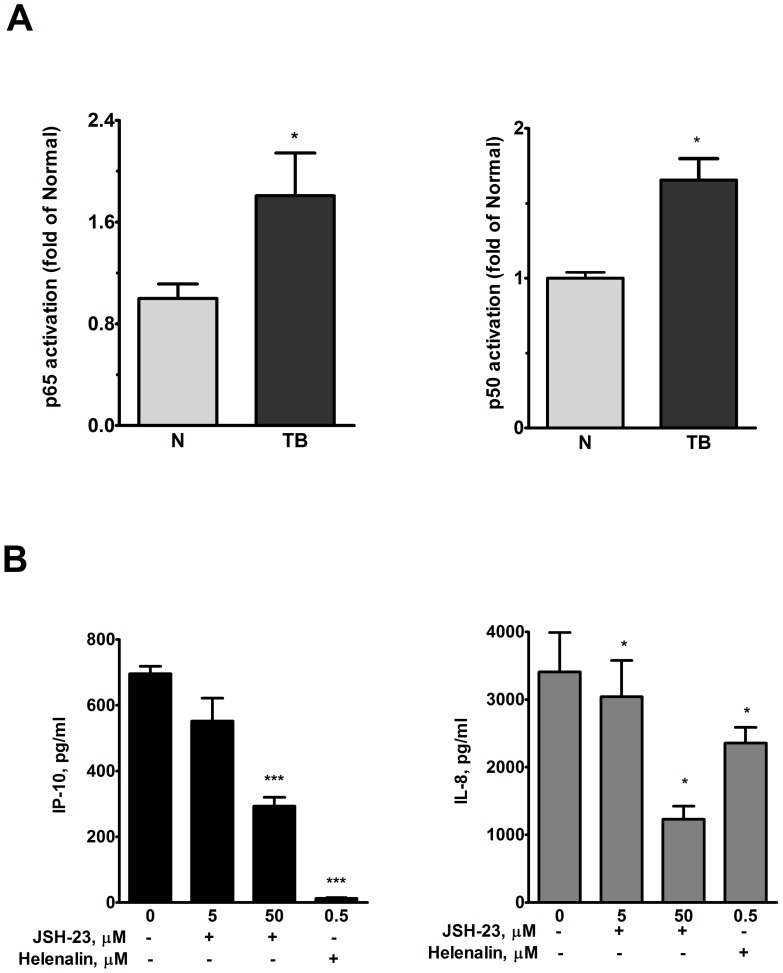
Increased NF-κB subunits, release of IP-10/CXCL10 and IL-8/CXCL8 in pulmonary TB. TransAM assay to measure the NF-κB subunit p65 and p50 activities reveals (A) an increase in basal state both p65 and p50 activities in PBMC of pulmonary TB patients (n = 5) compared with those of normal subjects (n = 4), * p<0.05 compared with normal subjects; (B) the release of IP-10/CXCL10 (Left panel) and IL-8/CXCL8 (Right panel) from PBMC of TB patients (n = 5) is significantly inhibited by NF-κB inhibitors either JSH-23 (5 and 50 μM) or Helenalin (0.5 μM) compared to those of the vehicle (0 μM). Data are means ± S.E. *p<0.05, ***p<0.001 compared to the vehicle.

Patients with active pulmonary TB had a higher serum level of IP-10/CXCL10 (392.2±70.5 pg/ml, n = 19) and IL-8/CXCL8 (31.9±8.5 pg/ml, n = 19) than that of normal subjects (62.8±5.4 pg/ml; 4.5±.0 pg/ml, n = 15, p<0.01, respectively) (Table 1). After 24 h of culture, PBMC from TB patients released higher levels of IP-10/CXCL10 (278.5±54.6 pg/ml, n = 19) and IL-8/CXCL8 (27639.0±3368.0 pg/ml, n = 19) than those of normal subjects (29.6±5.5 pg/ml; 6917.0±1900.0 pg/ml, n = 15, p<0.01, respectively) (Table 1). After 24 h of culture, AM from patients with TB (n = 19) also released a greater amount of IP-10/CXCL10 (950.9±283.0 pg/ml) and IL-8/CXCL8 (37834.0±4208.0 pg/ml) than those of normal subjects (30.0±7.6 and 10414.0±2683.0 pg/ml, p<0.01, n = 15, respectively) (Table 1). The retrieved cells in bronchoalveolar lavage fluid or mononuclear cells in the peripheral blood were not enough to do all the experiments in some patients. Therefore, the data of the following experiments were not available from all the studied subjects. TransAM assay revealed an increase in NF-κB p65 and p50 subunits in the PBMC of TB patients (Figure 2A). PBMC from TB patients treated with a specific NF-κB inhibitors, JSH-23 (50 µM) and Helenalin (0.5 µM), suppressed the release of IP-10/CXCL10 (293.2±26.9 pg/ml and 12.5±5.5 pg/ml, respectively, p*<*0.001) and IL-8/CXCL8 (2355.0±232.8 pg/ml and 2355±232.8 pg/ml, respectively, p*<*0.05) than those of control (695.9±22.6 and 4435.0±215.6 pg/ml n = 5, respectively) (Figure 2B), indicating the release of IP-10/CXCL10 and IL-8/CXCL8 is mediated via NF-κB. There was no significant cytotoxicity induced by JSH-23 either by 5 µM or 50 µM (cell viability >96%).

### Increased expression of IP-10/CXCL10 and IL-8/CXCL8 mRNA in pulmonary TB

**Figure 3:**
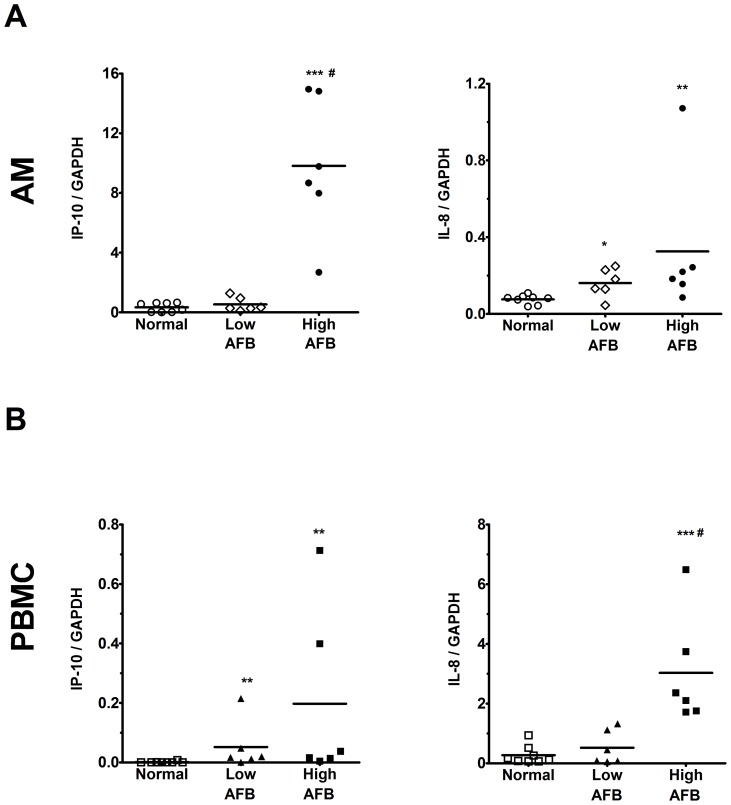
Expression of IP-10/CXCL10 and IL-8/CXCL8 mRNA in AM and PBMC of TB patients. The mRNA levels of IP-10/CXCL10 (left panel) and IL-8/CXCL8 (right panel) in (A) alveolar macrophages (AM) and (B) PBMC of TB patients with high bacterial load (High AFB, n = 6) are significantly higher than that of low bacterial load TB patients (Low AFB, n = 6) and normal subjects (Normal, n = 8). Values were normalized by GAPDH. Data are means ± SE. *p<0.05, ** p<0.01, *** p*<*0.001 compared with normal subjects. # p<0.01 compared with low bacterial load TB patients.

Levels of IP-10/CXCL10 and IL-8/CXCL8 mRNA were higher in AM from patients with AFB-high active pulmonary TB (9.8±1.9, p*<*0.01 and 0.3±0.2, p*<*0.01, n = 6, respectively) than those of normal subjects (0.3±0.1 and 0.1±0.01, n = 8, respectively) (Figure 3A). Patients with AFB-low active pulmonary TB had a higher level of IL-8/CXCL8 mRNA (0.2±0.03, n = 6, p*<*0.05), but not IP-10/CXCL10 mRNA (0.5±0.2, n = 6) than normal subjects (Figure 3A). The levels of IP-10/CXCL10 and IL-8/CXCL8 mRNA from PBMC were significantly higher in patients with AFB-high active pulmonary TB (0.2±0.1, n = 6, p*<*0.01 and 3.0±0.8, n = 6, p*<*0.001, respectively) as compared with normal subjects (0.001±0.001 and 0.3±0.1, n = 8, respectively) (Figure 3B). Patients with AFB-low active pulmonary TB had a higher level of IP-10/CXCL10 mRNA (0.05±0.03, n = 6, p<0.01), but not IL-8/CXCL8 (0.5±0.2, n = 6) than normal subjects (Figure 3B). A trend for bacterial load-associated increase in IP-10/CXCL10 and IL-8/CXCL8 was also demonstrated by post-test for linear trend compared with normal subjects (p<0.05).

### Expression of NRF in AM and PBMC

**Figure 4:**
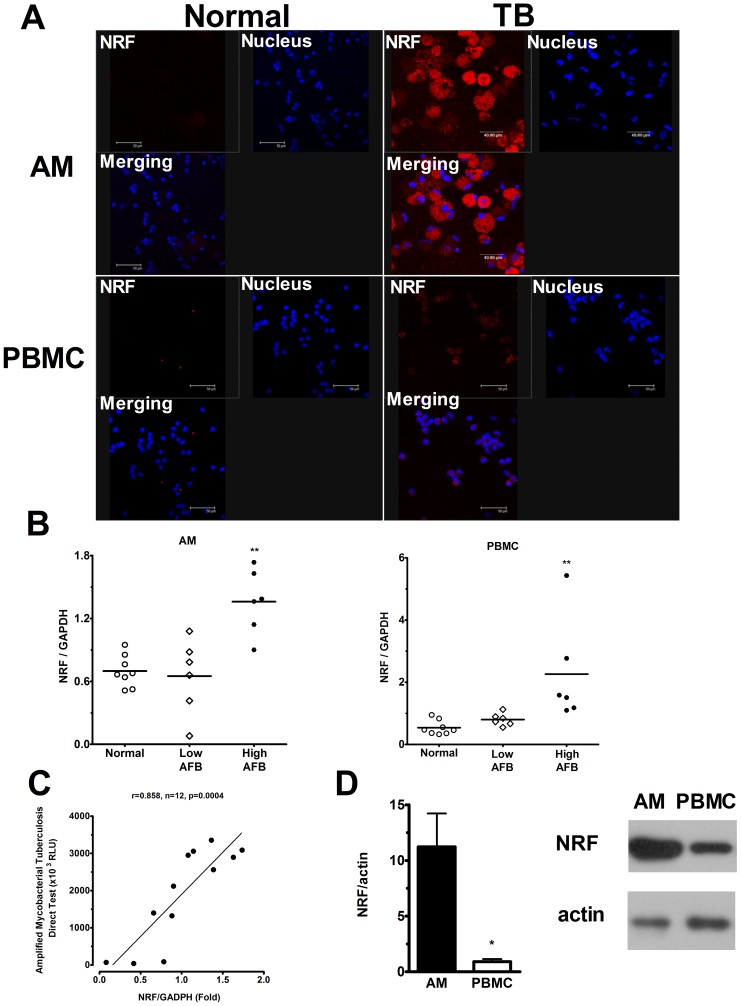
Expression of NRF in AM and PBMC in normal subjects and TB patients. (A) The expression of NRF in alveolar macrophages (AM) and PBMC in normal subjects (Left panel) and pulmonary TB patients (Right panel) by confocal image analysis. AM and PBMC were labeled by anti-NRF-cy3 and Hoechst dye. (B) The expression of NRF mRNA in AM (Left panel) and PBMC (Right panel) of patients with high bacterial load (High AFB, n = 6) is significantly higher than those of TB patients with low bacterial load (Low AFB, n = 6) or normal subjects (Normal, n = 8). (C) The intracellular load of *M. TB* in AM in terms of ribosomal RNA (AMTD response) is significantly positively correlated to the NRF expression levels in AM of TB patients. (D) The level of NRF expression in TB patients was significantly higher in AMs compared to the corresponding PBMCs. Data are means ± SE. * p<0.05, ** p<0.01.

Confocal microscopic analysis revealed an increased expression of NRF either in AM or in PBMC of TB patients (Figure 4A). Quantification by quantitative RT-PCR demonstrated that NRF in PBMC was 4.2-fold in AFB-high group (2.3±0.7 folds, n = 6, p<0.01) and 1.5-fold in AFB-low group (0.8±0.1 folds, n = 6) as compared with that in normal subjects (0.5±0.1 folds, n = 8) (Figure 4B). In AM, NRF was 1.8-fold in AFB-high group (1.4±0.2 folds, n = 6, p<0.01) and 0.9-fold in AFB-low group (0.7±0.2 folds, n = 6) as compared with normal subjects (0.7±0.1 folds, n = 8) (Figure 4B). Post-test for linear trend revealed a trend of bacterial load-associated increase of NRF in active pulmonary TB patients compared with normal subjected (p<0.05). The intracellular load of *M. TB* in AM in terms of ribosomal RNA (AMTD response) was significantly positively correlated to the NRF expression levels (r = 0.858, n = 12, p = 0.0004) (Figure 4C). However, the AMTD test showed negative result in PBMCs of patients with pulmonary TB. The level of NRF expression in TB patients was significantly higher in AMs compared to the corresponding PBMCs (Figure 4D).

### NRF suppresses the IP-10/CXCL10 and IL-8/CXCL8 promoters in PBMC

**Figure 5:**
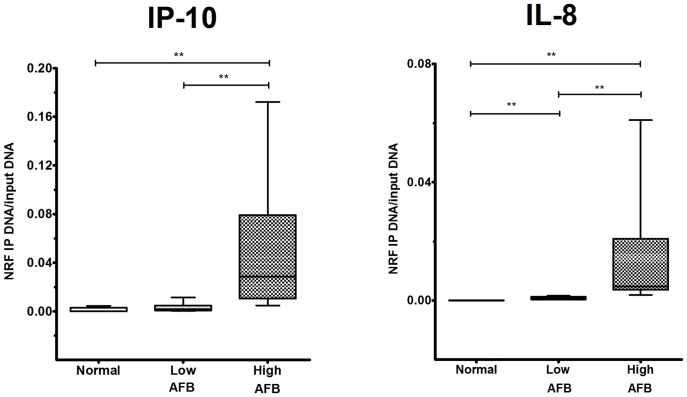
NRF binding to IP-10/CXCL10 and IL-8/CXCL8 promoter sites by ChIP assays. ChIP assays were performed on PBMC from active pulmonary TB patients and normal subjects by using a NRF antibody. The IP-DNA was quantified by q-PCR with primer pairs specific to IP-10/CXCL10 and IL-8/CXCL8 NRE sites at the promoters. Values were normalized by input DNA. The ChIP assays showed a higher binding of NRF to IP-10/CXCL10 (left panel) and IL-8/CXCL8 (right panel) promoter sites in TB patients with high bacterial load (High AFB, n = 6) compared to those of TB patients with low bacterial load (Low AFB, n = 6) or normal subjects (Normal, n = 5). Data are means ± SE. ** p<0.01.

By binding to the NRE in the IL-8/CXCL8 promoter, NRF suppresses its basal transcription [27]. There is a similar specific NRE sequence in the promoter site of IP-10/CXCL10 (Table 2). To this end, ChIP analysis with the antibody specific for NRF was used. The amount of IP-DNA was determined by RT-qPCR using primer pairs (Table 3) amplifying a region around the NRE site in the IL-8/CXCL8 or IP-10/CXCL10 promoter. To demonstrate the site specificity of the assay, a primer pair amplifying an irrelevant site around the 3′-UTR was also used. IgG controls were used to demonstrate the specificity of the antibody. In the PBMC of normal subjects, a low but consistently detected enrichment of IP-DNA over background (the IgG control) was observed (IP-10/CXCL10: 0.001±0.001, n = 6, and IL-8/CXCL8: 0.001±0.001, n = 6, respectively) (Figure 5). In the PBMC of AFB-high patients, the amount of IP-DNA at IP-10/CXCL10 and IL-8/CXCL8 promoters was 21.8-folds (0.02±0.01, n = 6; p*<*0.05) and 6.4 folds (0.009±0.007, n = 6; p*<*0.05) that of normal subjects (Figure 5). In contrast, changes of the IP-DNA were not seen when the primers for 3′-UTR were used (data not shown). Up-regulation of NRF in the PBMC of active pulmonary TB patients with high bacterial load is associated with increased occupancy of NRF at the IP-10/CXCL10 and IL-8/CXCL-8 promoters and decreased the synthesis of corresponding mRNA.

**Table 2:** Sequence comparison of the NRE sites in IFN-β, IL-8/CXCL8, hiNOS, IP-10/CXCL10 promoters.

Construct Gene	NRE sequence
IFN-β promoter	(−60) AATTCCTCTGA (−50)
IL-8 promoter	(−1415) AATTCCTCTGA (−1405)
hiNOS promoter	(−6749) AATTCCTCAGC (−6739)
IP-10 promoter	(−503) AACTCCTGAGC (−493)

**Table 3:** Transcript and sequence of each primer used in real time RT-PCR and ChIP assays.

Transcript	Primer Sequence
**For RT-PCR**	
IP-10	F: 5′-AGTTAGCAAGGAAAGGTCT-3′
	R: 5′-ACATTATAGTGCCAGGT-3′
IL-8	F: 5′-AGATCTGAAGTGTGATGACTCAGG-3′
	R: 5′-GAAGCTTGTGTGCTCTGCTGTCTC-3′
NRF	F: 5′-AGAAAGATGGGTTGGACT-3′
	R: 5′-CTGTGTGGCTCTCGGA-3′
GAPDH	F: 5′-TTCCAGGAGCGAGATCCCT-3′
	R: 5′-CACCCATGACGAACATGGG-3′
**For ChIP assays**	
NRF promoter	F: 5′-AGGTTCAAGCAGTTTTCC-3′
	R: 5′-CTGTAATCTCAGCACTTTGG-3′
IP-10 promoter	F: 5′-AGGCTGGTCTCAAACT-3′
	R: 5′-CCTCCCACATCCAATTACT-3′
IL-8 promoter	F: 5′-GGGCCATCAGTTGCAAATC-3′
	R: 5′-TTCCTTCCGGTGGTTTCTTC-3′
IL-8 3′-untranslated region (3′-UTR)	F: 5′-AGGTTCAAGCAGTTTTCC-3′
	R: 5′-CTGTAATCTCAGCACTTTGG-3′
IP-10 3′-untranslated region (3′-UTR),	F: 5′- TTGAGTTATAATTACTTAT-3′
	R: 5′-TGAAAAGAAGGGTGAGAAGAG-3′

*Abbreviations*: F =  forward; R =  reverse; IP-10 =  Interferon gamma-induced protein-10; IL-8 =  interleukin-8; NRF  =  NF-κB repressing factor; ChIP  =  chromatin immunoprecipitation.

### NRF related to release of IL-8/CXCL8 and IP-10/CXCL10 in AM and PBMC

**Figure 6:**
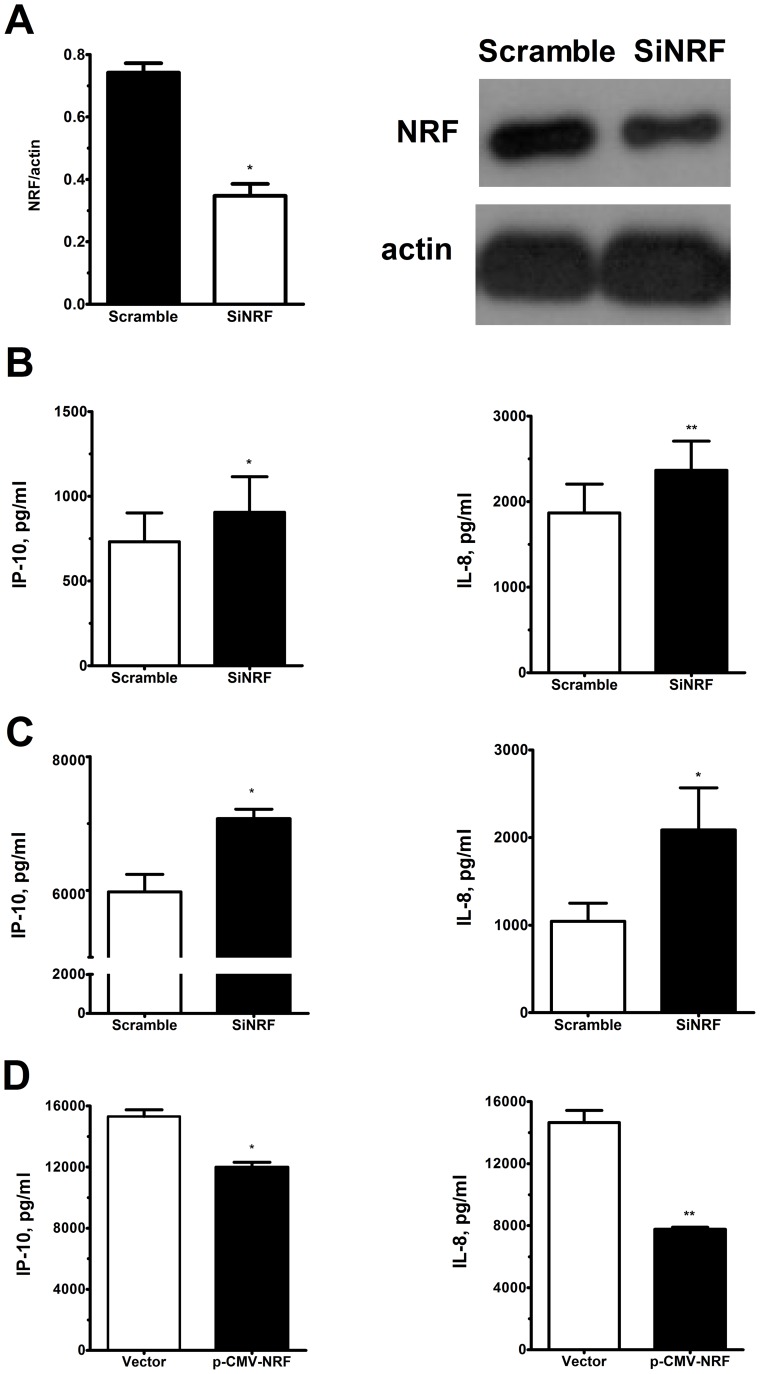
Inhibition of NRF increased the release of IP-10/CXCL10 and IL-8/CXCL8 from PBMC of TB patients. (A) Transfection with siRNA targeting NRF (SiNRF) or scramble control (Scramble) for 48 hours significantly decreased the expression of NRF protein in PBMCs of TB patients. (B) SiNRF transfection (SiNRF) significantly increases the release of IP-10/CXCL10 and IL-8/CXCL8 from PBMCs of TB patients with high bacterial load (n = 4) compared to scramble control (Scramble). (C) PBMC of normal subjects (n = 4) were transfected with SiNRF or Scramble for 48 hours, and then incubated with heated TB 5 μg/ml for 6 hours. Transfection with SiNRF significantly increased heated TB-induced release of IP-10/CXCL10 and of IL-8/CXCL8. (D) PBMC of normal subjects (n = 4) were transfected with NRF plasmid or vector for 24 hours, and then incubated with heated TB 5 μg/ml for 6 hours. Overexpression of NRF significantly decreased the heated TB-induced release of IP-10/CXCL10 and IL-8/CXCL8 from PBMC. Data are means ± S.E. * p<0.05, ** p<0.01.

We conducted *in vitro* experiments of NRF knockdown by the siRNA targeting NRF (siNRF) or non-targeting siRNA (scramble RNA, siCONTROL) with PBMC from AFB-high patients. PBMC were transfected with siNRF or siCONTROL for 48 h. The expression of NRF was significantly inhibited by 53.4±4.4% after targeting siNRF compared to siCONTROL (Figure 6A). The transfected PBMCs were further cultured for 6h, and the culture media were harvested for ELISA assay. PBMC transfected with siNRF released a greater levels of IP-10/CXCL10 (905.9±209.3 pg/ml, n = 6, p*<*0.05) and IL-8/CXCL8 (2365±340.8 pg/ml, n = 6, p*<*0.01) when compared with those transfected with siCONTROL (Figure 6B). Although the increase in such extents of IP-10/CXCL10 and IL-8/CXCL8 after transfection with siNRF may not reach biological significance, the results confirm the role of NRF in inhibition of IP-10/CXCL10 and IL-8/CXCL8. The significance of this experiment was limited by the relatively low transfection efficacy of siNRF in primary human cells that have been upregulated in active pulmonary TB infection. MTT assays showed similar cellular viability between siNRF and scramble siRNA transfected cells (data not shown). Those data indicate that endogenous NRF in PBMC suppresses basal production of IP-10/CXCL10 and IL-8/CXCL8 in AFB-high patients.

To delineate the mechanism underlying the up-regulation of NRF in active pulmonary TB, PBMC from normal subjects were exposed to H. TB. PBMC of normal subjects when exposed to H. TB (2.5, 5 and 10 μg/ml), dose-dependently increased the release of IP-10/CXCL10 and IL-8/CXCL8 proteins with a concomitant increase in IP-10/CXCL10, IL-8/CXCL8 and NRF mRNA synthesis (data not show). Pretreated with a NF-κB inhibitor JSH-23 (50 µM) before exposure to H. TB suppressed the release of IP-10/CXCL10 and IL-8/CXCL8 (62.6±22.7 pg/ml, p<0.01 and 17570.0±1969.0 pg/ml, p*<*0.05, n = 6, respectively) than control (181.5±29.7 pg/ml and 24927.0±2465.0 pg/ml, respectively). SiNRF transfected PBMCs of normal subjects, when exposed to H. TB, induced a higher level of IP-10/CXCL10 and IL-8/CXCL8 protein release, when compared with siCONTROL (Figure 6C). Forced expression of NRF in PBMCs of normal subject, then exposed to H. TB, decreased IP-10/CXCL10 and IL-8/CXCL8 protein release, when compared with vector controls (Figure 6D). Those observations suggest that the intracellular level of NRF regulates the production of IP-10/CXCL10 and IL-8/CXCL8 from PBMC in TB infection.

## Discussion

We have demonstrated NRF expression is up-regulated in AM and PBMC of active pulmonary TB patients with high bacterial load (AFB-high group). Although the bacterial loads in AMs in terms of ribosomal RNA were highly correlated with the NRF expression levels in AM, the absence of *M. TB* ribosomal RNA in PBMCs indicates AM or PBMCs of TB patients do not necessarily contain *M. TB* bacilli to express NRF or to release IP-10/CXCL10 or IL-8/CXCL8. AM or PBMCs might be stimulated by bacterial products directly or released into circulation when PBMCs passed through the pulmonary circulation. This postulation is confirmed by our in vitro studies on the effect of H. TB on NRF expression, release of IP-10/CXCL10 and IL-8/CXCL8 from PBMCs and AM retrieved from normal subjects. The increased level of NRF was found associated with an increased occupancy at the promoter sites of IP-10/CXCL10 and IL-8/CXCL8 by ChIP assay. Knockdown of NRF by NRF siRNA augmented the mRNA synthesis and protein release of both IP-10/CXCL10 and IL-8/CXCL8 from PBMC of active pulmonary TB patients with high bacterial load. Our results indicate a pathophysiological role of NRF in repression of IP-10/CXCL10 and IL-8/CXCL8 synthesis by AM and PBMC in active pulmonary TB.

The mechanism responsible for the recruitment of leukocytes to the site of inflammation in pulmonary tuberculosis involves the local generation of chemokines and immune regulation to the injured site [31]. C-X-C chemokines, such as IP-10/CXCL10 and IL-8/CXCL8, are predominantly involved in neutrophil, lymphocyte and monocyte recruitment [31], [32]. IP-10/CXCL10, which is induced either by IFN-γ, IFN-β or virus [33] has been reported to increase in pulmonary TB [19], [34], [35], which we also observed. MTb infection stimulates the secretion of IP-10/CXCL10 that preferentially attracts Th1- and Tc1-activated lymphocytes and NK cells through CXCR3 [16]–[18], and may play a role in generation and function of effector T cells by promoting antigen-specific proliferation and IFN-γ secretion [18], [36], [37]. IL-8/CXCL8 is involved in monocyte, lymphocyte, and neutrophil recruitment that constitutes innate immunity against Mtb infection [38], [39], and implicated in granuloma formation and maintenance in TB [12], [40], [41]. Thus, IP-10/CXCL10 and IL-8/CXCL8 play an important role in the innate immune response and bridges to the adaptive cellular response. The up-regulated release of IP-10/CXCL10 and IL-8/CXCL8 in patients with active pulmonary TB was suppressed by specific NF-κB inhibitor JSH-23 [42], [43], suggesting both IP-10/CXCL10 and IL-8/CXCL8 release is mediated through NF-κB activation.

NRF binding to DNA specifically abolishes the transcriptional activity of the bordering NF-κB-binding sites by a non-competing, distance and position-independent mechanism [26]. NRF plays a dual role in IL-1-induced IL-8/CXCL8 transcription [27]. The regulatory mechanism for NRF synthesis is not yet elucidated. In our previous study, human neutrophil elastase induced NRF synthesis and recruitment to IL-8/CXCL8 promoter site, where NRF inhibited NF-κB transactivating activity or directly suppress the promoter [44]. In the present study, the expression of NRF protein and mRNA was highly increased in AM and PBMC of pulmonary TB patients with high bacterial load. ChIP assay was performed to examine whether the up-regulated NRF increases recruitment to IL-8/CXCL8 and IP-10/CXCL10 promoters to confer the repressive effect. There is a potential NRE site (AACTCCTGAGC, -503 to −493 from the translation start site, HGNC ID: 10637, Table 2) upstream to the translation start site in the IP-10/CXCL10 gene with 81.8% similarity to hiNOS NRE site [25]. This suggests a homology in regulation of both iNOS and IP-10/CXCL10 genes by NRF. Thus, the primer pairs to amplify a region around the NRE site in the IP-10/CXCL10 promoter were also used in our ChIP assay. There was an increase in NRF occupancy at IP-10/CXCL10 and IL-8/CXCL8 promoter sites in the PBMC of AFB-high patients, suggesting an inhibition at the transcriptional initiation. The binding of NRF to IP-10 or IL-8 promoter sites is not convincing enough to answer for the repressive mechanism. There is a need of future detailed mechanistic experiment to explore the epigenetic mechanisms. Nevertheless, the evidence for NRF binding to IP-10 and IL-8 promoter sites in PBMCs of TB patients provides a link between NRF expression and its repressive effect on IP-10 and IL-8 synthesis, and may be through what we have recently reported the epigenetic modulation mechanism of NRF in PBMCs of COPD patients [45]. The functional role of NRF was further confirmed by knockdown of NRF by RNA interference to augment IP-10/CXCL10 and IL-8/CXCL8 release from PBMC of AFB-high patients.

It is possible that up-regulation of NRF may serve as an endogenous repressor to prevent extensive tissue responses resulting from a robust increase in IP-10/CXCL10 and IL-8/CXCL8 release when bacterial load is high. However, the levels of IP-10 in bronchoalveolar lavage cells are negatively related to cavity formation in active pulmonary TB patients. IP-10 and IL-6 expression above median reduces the odds of cavities by around 80% [46]. Since IP-10/CXCL10 is involved in trafficking and activation of Th1 lymphocytes [17], and IL-8/CXCL8, in addition to chemotaxis, also reduces the survival of MTb within infected macrophages [13], the up-regulation of NRF in AM and PBMC of active pulmonary TB patients with high bacterial load to repress IP-10/CXCL10 and IL-8/CXCL8 release may render MTb to attenuate host innate immunity attacks resulting in prolonged survival and increased proliferation.

The mechanism underlying up-regulation of NRF in active pulmonary TB is yet to be determined. NRF synthesis was induced by direct exposure of monocytes of healthy subjects to heated TB bacilli, suggesting the induction of NRF synthesis might be directly related to exposure to TB bacilli itself or its component. MTb and its components have been reported to cause a constitutive degradation of IκB-α, leading to NF-κB activation in monocytes from TB patients [47]. Our previous study has indicated that NF-κB activation induces NRF synthesis through NF-κB subunit p65 binding to the NRF promoter to transcriptionally activate NRF mRNA synthesis [40]. In the present study, the expression of NF-κB p65 and p50 subunits increased in the PBMC of TB patients. Whether a similar mechanism also applies to MTb induced NRF synthesis deserves further study.

The distinction between AFB-high and AFB-low is arbitrary. All the patients in AFB-high group had sputum AFB smear of grade 2 or greater on a single day. In contrast, patients in AFB-low group had sputum AFB smear of grade 1 or less on any single day. To avoid sputum sampling errors or inconsistency, the grading over 3 days were summed as an index of bacterial sputum load. This arbitrary distinction excludes patients with AFB smear of grades in-between these two groups. The marked differences in NRF, CXCL10/IP-10 and CXCL8/IL-8 between these two groups are hardly attributed to the difference in bacterial load. However, a previous study has demonstrated that the number and maximum size of cavities and involved lobes measured by computed tompgraphy significantly differed between sputum AFB 2+ or greater Group compared to the other groups (negative or doubtful, or sputum smear AFB 1+) [48]. Thus, it is possible that the host response to mycobacterial infection may be exaggerated when bacterial load exceeds to some extent.

In summary, we demonstrated that NRF, for the first time in human disease, is up-regulated in the PBMC and AM of active pulmonary TB patients with high bacterial load to repress MTb induced IP-10/CXCL10 and IL-8/CXCL8 synthesis and protein release. Thus, NRF may play a role in modulation of innate immunity and subsequent adaptive immunity against MTb infection.
